# *Dnah9* mutant mice and organoid models recapitulate the clinical features of patients with PCD and provide an excellent platform for drug screening

**DOI:** 10.1038/s41419-022-05010-5

**Published:** 2022-06-21

**Authors:** Rui Zheng, Wenhao Yang, Yuting Wen, Liang Xie, Fang Shi, Danli Lu, Jiaxin Luo, Yan Li, Rui Zhang, Ting Chen, Lina Chen, Wenming Xu, Hanmin Liu

**Affiliations:** 1 Department of Obstetrics/Gynecology, Joint Laboratory of Reproductive Medicine (SCU-CUHK), Key Laboratory of Obstetric, Gynecologic, and Pediatric Diseases and Birth Defects of Ministry of Education, West China Second University Hospital, Sichuan University, Chengdu, China; 2 Department of Pediatric Pulmonology and Immunology, West China Second University Hospital, Sichuan University, Chengdu, China; 3 Key Laboratory of Chronobiology (Sichuan University), National Health Commission of China, Chengdu, China; 4 The Joint Laboratory for Lung Development and Related Diseases of West China Second University Hospital, Sichuan University and School of Life Sciences of Fudan University, West China Institute of Women and Children’s Health, West China Second University Hospital, Sichuan University, Chengdu, China; 5 Sichuan Birth Defects Clinical Research Center, West China Second University Hospital, Sichuan University, Chengdu, China; 6 Key Laboratory of Birth Defects and Related Diseases of Women and Children (Sichuan University), Ministry of Education, Chengdu, China

**Keywords:** Drug discovery, Molecular biology

## Abstract

Primary cilia dyskinesia (PCD) is a rare genetic disease caused by ciliary structural or functional defects. It causes severe outcomes in patients, including recurrent upper and lower airway infections, progressive lung failure, and randomization of heterotaxy. To date, although 50 genes have been shown to be responsible for PCD, the etiology remains elusive. Meanwhile, owing to the lack of a model mimicking the pathogenesis that can be used as a drug screening platform, thereby slowing the development of related therapies. In the current study, we identified compound mutation of *DNAH9* in a patient with PCD with the following clinical features: recurrent respiratory tract infections, low lung function, and ultrastructural defects of the outer dynein arms (ODAs). Bioinformatic analysis, structure simulation assay, and western blot analysis showed that the mutations affected the structure and expression of DNAH9 protein. *Dnah9* knock-down (KD) mice recapitulated the patient phenotypes, including low lung function, mucin accumulation, and increased immune cell infiltration. Immunostaining, western blot, and co-immunoprecipitation analyses were performed to clarify that DNAH9 interacted with CCDC114/GAS8 and diminished their protein levels. Furthermore, we constructed an airway organoid of *Dnah9* KD mice and discovered that it could mimic the key features of the PCD phenotypes. We then used organoid as a drug screening model to identify mitochondrial-targeting drugs that can partially elevate cilia beating in *Dnah9* KD organoid. Collectively, our results demonstrated that *Dnah9* KD mice and an organoid model can recapture the clinical features of patients with PCD and provide an excellent drug screening platform for human ciliopathies.

## Introduction

Primary cilia dyskinesia (PCD), a rare genetic ciliopathic disease, can cause severe outcomes in pediatric patients, including bronchiectasis, recurrent respiratory tract infections, nasosinusitis, and organ laterality defects. So far, fifty genes have been proved to be responsible for the clinical features of PCD, but its etiology remains elusive [1–4]. Cilia are evolutionarily conserved, hair-like microtubule-based organelles that protrude from the plasma membrane. Cilia are categorized into two groups according to ciliary motility as primary cilia and motile cilia. Structurally, the cilium is composed of the basal body, transition zone, axoneme, and ciliary membrane. Primary cilia are usually immotile and have a “9 + 0” axoneme structure, whereas most motile cilia have a “9 + 2” axoneme structure with a central pair of microtubule singlets inside the outer nine microtubule outlets.

In motile cilia, axonemal dyneins, including the inner dynein arm (IDA) and outer dynein arm (ODA) complexes, cooperate with radial spokes, nexin-dynein regulatory complexes, and the central pair of microtubules to gain ciliary motility. ODA mutations have been linked to a significant proportion of PCD mutations [5]. In mammalian motile cilia, there are two types of ODAs: type 1 ODA containing dynein axonemal heavy chain (DNAH) 5 and DNAH11, and type 2 ODA containing DNAH5 and DNAH9 [6, 7]. In particular, DNAH5 displays a pan-axonemal distribution along the ciliary axoneme; DNAH9 localizes to the distal axoneme, whereas DNHA11 localizes to the proximal compartment of the axoneme [6, 7]. Defective DNAH5 causes complete or nearly complete cilia immobility due to the absence of ODAs [8]. However, loss-of-function of DNAH11 causes no apparent impairment of the ciliary ultrastructure but can affect its beating pattern [9]. DNAH9 is located at distal cilium, whose mutation has been proved to associate with PCD [10, 11]. Furthermore, SNPs in *DNAH9* are related to early smoking, contributing to bronchial hyperresponsiveness [10]. Therefore, comprehensively investigating the function of *DNAH9* in ciliary development represents an excellent opportunity to understand the complex functions of ODA proteins in distal regions of motile cilia. Moreover, whether mutations in ODA genes such as *DNAH5* and *DNAH9* cause similar phenotypes and the relationship between different gene mutations and the variable clinical phenotypes of patients with PCD remain unclear. Furthermore, ODA is connected to the axoneme through a docking complex (DC), ODA-DC, including a coiled‑coil domain containing (CCDC) 114, CCDC151, and armadillo repeat-containing protein 4 (ARMC4) [12–14]. Nexin-dynein regulatory complex contains proteins such as growth arrest-specific 8 (GAS8), which also play a role in ODA assembly [15]. Therefore, dysfunction of ODA-associated protein can also induce PCD.

Biochemical and genetic studies have demonstrated that PCD pathogenesis is associated with ciliary structural protein dysfunction [16]. However, related therapies are developing slowly, as most studies have failed to mimic PCD pathogenesis. Isolation of primary cilia cells is difficult, so phenotypic studies are hard to conduct. Furthermore, considering that the tracheal tissue epithelium contains multiciliated, secretory, goblet, and basal stem/progenitor cells [17], the microenvironment and interactions of these cell types are critical for ciliary function [18]. Moreover, the paracrine secretion of cytokines, such as granulocyte-macrophage colony-stimulating factor (GM-CSF), monocyte chemoattractant protein-1 (MCP-1), and tumor necrosis factor-alpha (TNFα), also plays critical roles in PCD-related pathology [19]. Although zebrafish and mice are widely used as in vivo models for PCD gene identification [3, 20], the model establishment is time-consuming and expensive. In this regard, lung organoids have emerged as a promising system for bridging the gap between cell and in vivo models, for example, COVID-19 studies [21, 22]. Moreover, emerging studies have shown that repeated infection is a major characteristic of PCD; therefore, identifying the major cytokine changes during infections may help identify the critical immune factors in disease-related conditions [23, 24]. Notably, the role of the organoid model in PCD study and PCD drug screening remain unexplored, although lung organoids have been used in other fields of research [21, 25]. Recently, a study shows that mitochondria might also play a role in cilia growth [26]; therefore, drugs targeting mitochondria may represent novel candidates for screening new drugs to effectively rescue the PCD-related phenotypes.

In this study, we identified a compound heterozygous mutation of *DNAH9* in a patient with typical PCD and established *Dnah9* knock-down (KD) mice using the CRISPR/Cas9 method. Our results demonstrated that *Dnah9* KD mice phenocopied key PCD symptoms in the patient. We then investigated lung organoids from *Dnah9* KD mice and observed that the lung organoids displayed a PCD phenotype similar to that of the KD mouse model. Notably, we identified that drugs targeting energy metabolism are promising and effective in elevating ciliary beating in the PCD mouse organoid model. Our findings provide proof-of-concept that mouse lung-derived organoids can be an effective drug screening platform in the PCD field.

## Material and method

### Pedigree and patient information

A 7-year-old boy with recurrent respiratory tract infections and chronic rhinosinusitis and his parents were admitted for research at the West China Second University Hospital. Based on the ciliary structural defects detected by transmission electron microscopy (TEM), exome sequencing, and clinical symptoms, the patient was diagnosed with PCD. This study was approved by the Ethical Review Board of West China Second University Hospital, Sichuan University (approval number: 2020YFS0105). Informed consent was obtained from each participant.

### Determination of pulmonary function

To acclimatize the mice in the cavity, they were placed in a whole-body plethysmograph (WBP-4MR, TOW) for up to 30 min in advance, until the mouse calm down. The unrestrained male of WT and *Dnah9* KD mice were monitored for ≥20 min; each group contained three mice, replicating 3 thrice (*n* = 9). WT and Dnah9 KD mice were mixed to examine; researcher did not know the genotype of each mouse in advance. Tidal volume, breath frequency, inspiratory (Ti) and expiratory time (Te), maximum expiratory flow rate (PEF), maximum inspiratory flow rate (PIF), time to expire 65% of the “volume” (Rt), the ratio of time to PEF to Rt (Rpef), minute volume (Mv), respiration ratio (Volbal), enhanced pause (PenH), and mid-expiratory tidal flow (EF50) values were extracted, and whole-body plethysmograph (WBP) curves were depicted by the software (ResMass 1.4.2., TOW).

### *Dnah9* KD mice information

The animal experiments were approved by the Experimental Animal Management and Ethics Committee of West China Second University Hospital, Sichuan University. All animal procedures complied with the Animal Care and Use Committee of Sichuan University. *Dnah9* KD mice (C57BL/6 N) were generated using CRISPR/Cas9 technology and structure analysis, as described previously [27]. SgRNAs (sgRNA-1, 5′-CCACAGTGTGATGTACCGAGTCT-3′; sgRNA-2, 5′-AGGTCGGCTCCTGCCACACCAGC-3′) were synthesized by GenScript Co. Superovulated females (C57BL/6 N mice) were used for the production of oocytes. And zygotes with two pronuclei were microinjected with Cas9 and sgRNA. The zygotes were then transferred into the oviducts of the pseudopregnant mice (ICR). The coat-color difference between the strain of the embryo donor (C57BL/6 N) and the pseudopregnant recipient (ICR) indicated the newborn mice derived from the donor embryos. The founder mice and their offspring were genotyped by PCR using a Mouse Direct PCR Kit (Bimake, B40013) and sanger sequencing analysis. Founder mice were crossed and conceived 21 offspring, of which 11 heterozygous *Dnah9* mice were produced for next breeding. Heterozygous *Dnah9* mice and homozygous mice were used for the experiments. DNAH9 expression was examined using real-time PCR (RT-PCR) and western blot. The primers used for genotyping PCR are F 5′-ATCATCTGCAGGCAGGTGG-3′, R1 5′-ATTAAGTTTATATTTTGTACCTGCATGGAGC-3′, and R2 5′-CACACACATTGAATACAGGACATAGGG-3′.

### Protein structure prediction

The three-dimensional structure of DNAH9 was predicted using trRosetta [28], an accurate and fast de novo protein structure prediction software. The predicted structure models were molecular dynamically simulated for 50 ns using the GROMACS software [29]. Next, the predicted structure models were optimized using the Molecular Operating Environment (MOE) software, which involves repairing the incorrect number of hydrogen atoms, reducing clashes, and balancing the charge. Subsequently, the quality of the predicted structural models was evaluated by importing the structure on the ERRAT website (https://servicesn.mbi.ucla.edu/ERRAT/) [30]. Model 1, with the highest score, was selected as the final DNAH9 structural model by comparing the model structure quality scores. Furthermore, the harmfulness of the variant was analyzed using the PolyPhen-2 tool [31], SIFT, and Mutation Taster. The putative structural changes caused by the variant were predicted using the Missense3D online tool (http://www.sbg.bio.ic.ac.uk/~missense3d/) [32].

### Cilial-wave analysis of nasal cilia

For imaging nasal cilia generated flow, the nasal mucous membrane was taken from 40-day-old *Dnah9* KD and wild type (WT) control animals and placed in a 35-mm glass-bottomed culture dish with Dulbecco’s Modified Eagle Medium/Nutrient Mixture F-12 (DMEM/F12, Gibco). The dish was put under the 100× oil lens of a downright microscope (NIKON) and subjected to video microscopy recording at approximately 200 frames per second (fps). These videos were then used to measure ciliary beat frequency (CBF).

### Immunostaining and confocal analysis

The mouse airway organoid and nasal mucous membrane from *Dnah9* KD and WT mice were digested in 2.5% trypsin (Gibco) for 10 min and 20 min, respectively, and then transferred to a single-cell liquid medium and terminated with fetal bovine serum (FBS). Subsequently, the samples were smeared onto slides and fixed in 4% paraformaldehyde for 30 min. The slides were permeabilized using 0.5% Triton X-100, washed thrice with 1× phosphate-buffered saline (PBS), and blocked with 5% bovine serum albumin (BSA) for 1 h at room temperature (RT). The samples were then incubated with primary antibodies at 4 °C overnight. The next day, the slides were washed thrice and incubated with secondary antibodies labeled with Alexa Fluor 488 (Thermo Fisher, 1:1000) or Alexa Fluor 594 (Thermo Fisher, 1:1000) for 2 h at RT. Finally, the slides were counterstained with 4′, 6-diamidino-2-phenylindole (DAPI) for 5 min to label the nuclei. Images were acquired using a laser-scanning confocal microscope (Olympus). Quantification of the expression intensities of the different markers was performed using ImageJ software. The entire cilia region was defined to indicate the signal intensity. The method was adapted from a previous study [33].

### HEK293 cell culture and transfection

HEK293 cells were obtained from the American Type Culture Collection (ATCC). Cells were cultured in DMEM supplemented with 10% FBS (Thermo Fisher) and 1% penicillin and streptomycin at 37 °C. Expression plasmids encoding wild-type C-terminal fragments of *DNAH9* (c.5931–7131 and c.12235–13435) were synthesized by Vigene Biosciences (Jinan, China). The site-directed KOD-Plus-Mutagenesis Kit (Toyobo, SMK-101) was used to introduce missense mutation sites (c.6431 G > A, p. R2144Q; c.12835 G > A, p.G4279S). The pcDNA3.1-FLAG (+), WT-*DNAH9*, and mutant-*DNAH9* plasmids were transiently transfected with Lipofectamine 3000 (Invitrogen, L3000015) into HEK293 cells according to the manufacturer’s protocol. The site-directed mutation primers of the target sequences of *DNAH9* are c.G6431A-F 5′-GCTGGAGGAGCTCCTGGCTGTGCAGCACTCTGTATTTG-3′, c.G6431A-R 5′-TGCACAGCCAGGAGCTCCTCCAGCTGGACCACCTTG-3′; c.G12835A-F 5′-GCTCACTGAGGGAGCTGGAGCTCAGCTTAAAGGGGGAG-3′; c.G12835A-R 5′-TGAGCTCCAGCTCCCTCAGTGAGCGCTGAATCTC-3′

### Western blot

Transfected cells, mouse airway organoids, and lung tissues were lysed in ice-cold RIPA buffer (Beyotime, P0013C) containing inhibitor cocktail (Bimake, B14001) by ultrasonic waves and incubated on ice for 40 min. Next, samples were added to 5× sodium dodecyl sulfate (SDS) loading buffer and heated at 95 °C. Denatured proteins were separated on 3−8% Tris-acetate gel (Thermo Fisher, EA0375BOX) and transferred to a 0.45-µm pore-size polyvinylidene difluoride (PVDF) membrane (Millipore) at 40 V, overnight. Subsequently, the membranes were blocked in 5% skimmed milk for 1 h at RT and incubated with primary antibodies overnight at 4 °C. The following day, the samples were washed and incubated with goat anti-mouse IgG secondary antibody-HRP (1:5000, Thermo Fisher, 32230) and goat anti-rabbit IgG secondary antibody-HRP (1:5000, Thermo Fisher, 6120) in 5% skimmed milk at RT for 1 h. ECL chemical substrate (Millipore) was used to visualize the developed immunoblot.

### Co-immunoprecipitation (Co-IP)

The extracted proteins from mouse lung tissues were incubated with 3 µg of target antibodies and 3 µg rabbit IgG (ABclonal, AC005) overnight at 4 °C. Next, the mixture of each sample was added to a microcentrifuge tube containing 40 μl of prewashed Protein A/G magnetic beads (Invitrogen, 88802) and incubated for 2 h at RT with constant rotation. After being washed thrice with 1× PBS, the co-immunoprecipitated proteins were eluted with standard 2× SDS sample buffer and heated for 20 min at 70 °C. Finally, the co-immunoprecipitated proteins were separated on 10% sodium dodecyl sulfate-polyacrylamide gels for immunoblot analysis, as described earlier.

### RT-PCR

To assess the efficiency of *DNAH9-*knockdown, total RNA was isolated using TRIzol (Thermo Fisher, 10296010), chloroform, and isopropyl alcohol. cDNAs were prepared and amplified using a HiScript 1st strand cDNA Synthesis Kit (Vazyme, R111). RT-PCR was performed using an Applied Biosystems HT7900 with Power SYBR Green PCR Master Mix (Applied Biosystems, 4368706) following the manufacturer’s instructions. The primers are as follows: *Gapdh*-F, 5′-ACCCTTAAGAGGGATGCTGC-3′, *Gapdh*-R GTTCACACCGACCTTCACCA-3′; *Dnah9*-F 5′-GGCCCCACATGGTATGTCAA-3′, *Dnah9*-R 5′-CTGAGACTGGCTCACTGTGG-3′.

### Scanning electron microscopy (SEM) and TEM

For SEM, the tracheal samples from *Dnah9* KD and WT mice were fixed in 2.5% glutaraldehyde at 4 °C overnight. After the primary fixation, the samples were washed thrice in 1× PBS and post-fixed in 1% osmic acid for 1 h at 4 °C. The samples were then sequentially dehydrated in an ethanol series (30%, 50%, 75%, 95%, and absolute alcohol) for 10 min. Subsequently, sample drying was performed using a CO_2_ critical-point dryer (Eiko HCP-2) at RT. All dried specimens were glued to aluminum stubs, sputter-coated with an ionic sprayer meter (Eiko E-1020, Hitachi), and finally scanned with a field-emission SEM type Hitachi S3400.

For TEM, the samples were fixed in 3% glutaraldehyde and processed by the Chengdu Lilai Biomedicine Experiment Center following a conventional protocol. The final cilia of ultrathin tracheal epithelial cell sections were observed by TEM (TECNAI G2 F20).

### Antibody information

The antibodies used in western blot, Co-IP, and immunofluorescence staining (IF) analyses were anti-DNAH9 (WB:1:500, IF: 1:100; Thermo Fisher; PA5-45744), anti-LRRC6 (WB:1:1000; IF:1: 50; ORIGENE; TA806109), anti-CCDC114 (WB:1:100; IF:1:20; Santa Cruz; sc-398709), anti-GAS8 (WB:1:100; IF:1: 20; Santa Cruz; sc-390346), anti-GAPDH (WB: 1:5000; Abcam; ab8245), anti-CD68 (IF:1:200; Abcam; ab125212), anti-CD86 (IF:1:200; Abcam; ab238468), anti-CD45 (IF:1:100; Abcam; ab10558), and anti-acetyl-α-tubulin (IF: 1:200; CST; D20G3). anti-Krt5 (IF: 1:200; Abcam; ab52635), anti-CC-10 (IF:1:100; Santa Cruz; sc-365992); anti-Muc5AC (IF:1:200; Abcam; ab3649).

### Mouse airway organoid culture

Mouse airway tissue was isolated from 10-week-old WT and *Dnah9* KD mice. The solid primary airway was washed twice with cold AdDF + ++ buffer (Advanced DMEM/F12 containing 1× Glutamax, 10 mM HEPES, and antibiotics) and cut into 1 mm^3^ piece. These pieces were transferred into a 10 mL digestion medium containing 400 U/mL collagenase I (Sigma, 9001-12-1), 10 μM Y27632 (Selleck, S6390), and 10 U/ml DNAse I (Sigma, 10104159001) in AdDF + ++ for 1 h on a shaker at 37 °C. The digestion was sequentially terminated with 1 mL FBS (Gibco), and the tissue supernatant was sheared with 5 mL pipettes. After the shearing step, the supernatant was strained with a 40-μm strainer, centrifuged at 200 × g for 3 min, and discarded. Then, 1 mL red blood cell lysis buffer was added to the pellet for 3 min at RT to remove visible erythrocytes. Finally, the residual pellet was washed thrice with AdDF +++ and embedded in Matrigel (R&D, BME001). The suspension (30 μL) was seeded onto a single well of a 24-well plate and solidified at 37 °C for 15 min. Subsequently, 500 μL airway organoid culture medium was added to each well, and plates were incubated under standard culture conditions (37 °C, 5% CO_2_).

Mouse airway organoid culture medium consisted of AdDF + ++, 1× B27 (Gibco, 0080085SA), 5 mM nicotinamide (Sigma, N0636), 1.25 mM N-acetylcysteine (Sigma, A0737), 500 ng/mL R-spondin1 (R&D, 4645), 25 ng/mL recombinant human FGF7 (PeproTech, 450-61), 100 ng/mL recombinant human FGF10, 100 ng/mL recombinant human Noggin (R&D, 6057), 5 mΜ Y27632 (CST, 13624), 500 nM SB202190 (Selleck, S1077), and 500 nM A-8301 (Selleck, S8301). The culture medium was changed every 4 days.

Organoids were passaged every 2 weeks. All the organoids and Matrigel were resuspended in 1 mL TrypLE™ Select (1×) (Gibco, 0040090DG), incubated for 5–10 min at 37 °C, and were mechanical sheared with 1 ml pipettes. After washing thrice with AdDF + ++, the organoid pellets were resuspended in matrigel and reseeded in another well. Single cells of the digestion supernatant were seeded at 3000–4000 cells per well on a 24-well plate.

### Drug screening

*Dnah9* KD mouse airway organoids were digested into single cells, resuspended with matrigel, and reseeded to 1000 cells in 48-well plates. Upon gelation, 250 μL culture medium containing different drug concentrations was added to each well. Each test group was tested in four replicates, and another four wells were added culture medium with the same volume of PBS or DMSO (dimethylsulfoxide) as the control group. ATP was obtained from Sigma, and nicotinamide adenine dinucleotide (NADH) was purchased from Shanghai Yuanye Bio-Technology Co., Ltd. All other drugs were obtained from a validated company.

### Polyinosinic: polycytidylic acid [poly(I:C)] treatment

Poly (I:C) is a synthetic double-stranded RNA (dsRNA) that can elicit the secretion of type I interferon (IFN) and pro-inflammatory cytokines and is commonly used for immune activation both in vitro and in vivo. *Dnah9* KD and WT mouse airway organoids were digested and reseeded in 24-well plates at 3000 cells per well. A culture medium containing 100 ng/mL poly (I:C) (Sigma, 31852) was added to *Dnah9* KD and WT organoids after being cultured for 12 days, whereas the control group was treated with PBS. Each group consisted of three replicates. The supernatant and organoids were harvested after four days of poly (I:C) treatment.

### Cytokine array measurement

The organoid culture supernatant was collected after 4 days of intervention with poly (I:C) and then centrifuged to remove debris. The supernatant was assayed using the Mouse High Sensitivity T Cell Magnetic Bead Panel (Millipore, MHSTCMAG-70K) according to the manufacturer’s instructions.

### High-speed microscopy analysis for *Dnah9* KD mouse airway organoid

To record CBF, *Dnah9* KD and WT mouse airway organoids were prepared at RT (25 °C) for video microscopy with a 40× objective (Sprinter-HD Optronics). Movies were recorded at 200 fps and analyzed blindly by two researchers as described earlier. The CBF was recorded using video analysis.

### Statistical analysis

Every experiment was replicated at least thrice in the laboratory. Data are expressed as mean (*n* < 10) and mean ± standard deviation of the mean (*n* ≥ 10). All statistical analyses were performed using the GraphPad Prism software (version 8.00). Normality test to examine whether all statistical data adjusted to normal distribution. To compare the differences between the two groups, *T*-tests were used. When the data were not normally distributed, Mann–Whitney–Wilcoxon test was used instead of *T*-test to compare the difference between groups. A one-way analysis of variance (ANOVA) was used for statistical comparison to compare three or more groups. Significant differences between groups are represented by *P < 0.05.

## Results

### The patient harboring a novel compound mutant of *DNAH9* shows typical PCD lung pathophysiology

**Fig. 1:**
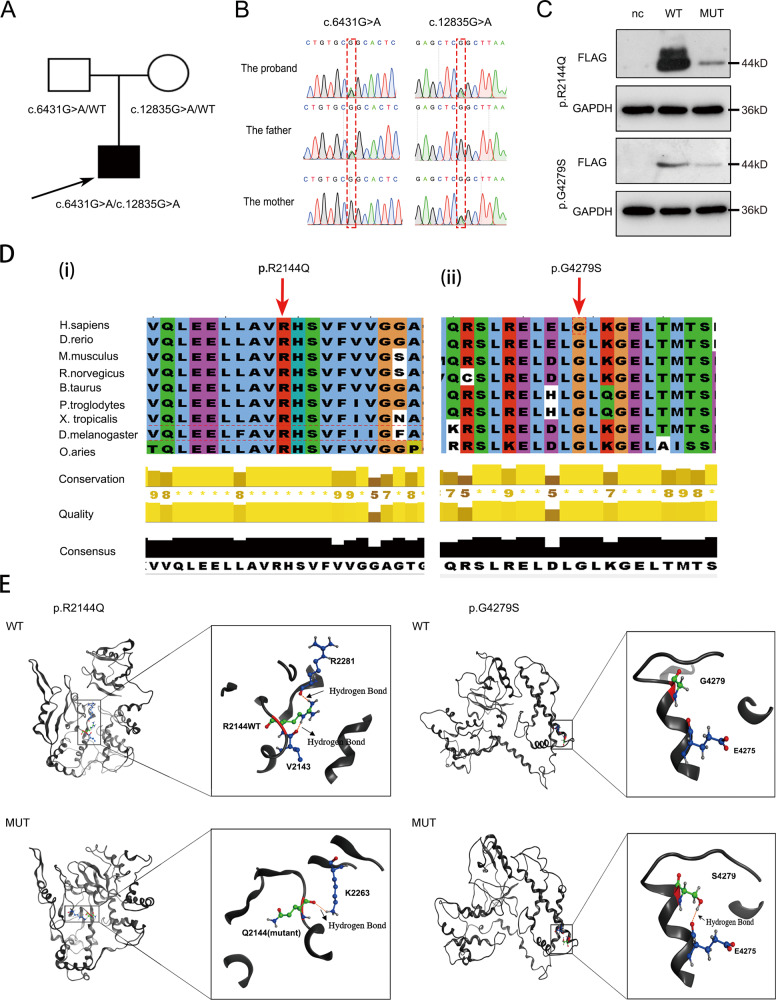
Compound heterozygous mutations in *DNAH9* reduce protein itself expression. **A** Family pedigree structure of the patient. The black squares represent the proband. **B** Sanger sequencing identifying the compound mutations in the patient and mutation carrier parents. **C** Western blot analysis of the *DNAH9* fragment in HEK293 cells transfected with wild type and mutant plasmids. GAPDH served as a loading control. **D** Multiple sequence alignment of the mutated amino acid site in DNAH9 protein in different species. Red arrows indicate the position of the variants. **E** The protein three-dimensional structural alteration of p.R2144Q and p.G4279S variants. The R2144 and Q2144 residues are shown in green, R2144 and its adjacent V2143/R2281residue form a hydrogen bond (yellow dotted line), respectively, whereas Q2144 form an aberrant hydrogen bond with K2263. The G4279 and S4279 residues are shown in green. G4279 do not form a hydrogen bond with other residues in wild type, whereas mutant S4279 can form a hydrogen bond with E4275.

In this study, we used exome sequencing to identify candidate genes related to PCD pathogenesis. Exome sequencing results showed that a 7-year-old boy had compound missense mutations of *DNAH9*, including c.6431 G > A (p.R2144Q) and c.12835 G > A (p.G4279S). The sequencing results from his parents demonstrated that the c.6431 G > A and c.12835 G > A mutations were inherited from his father and mother, respectively (Fig. 1A, B). Both mutations were rare or not mentioned in public databases, such as the ExAC Browser, GenomAD, or the 1000 Genome Project. Prediction results of SIFT, PoyPhen-2, and Mutation Taster tools demonstrated that the two mutations were probably detrimental (Supplementary Table 1). To further test whether c.6431 G > A and c.12835 G > A mutations affect protein expression, we generated *DNAH9*-fragment expression constructs containing each missense mutation in vitro. The results demonstrated that both mutants resulted in reduced protein expression compared to that in wild-type *DNAH9* (Fig. 1C). Bioinformatics analysis showed that both amino acids were conserved among mammalian species (Fig. 1D). Furthermore, structural analysis was conducted to analyze both mutation sites. The results showed that the mutations altered the hydrogen bond formation (Fig. 1E), which might affect the stability of the DNAH9 secondary structure.

**Fig. 2:**
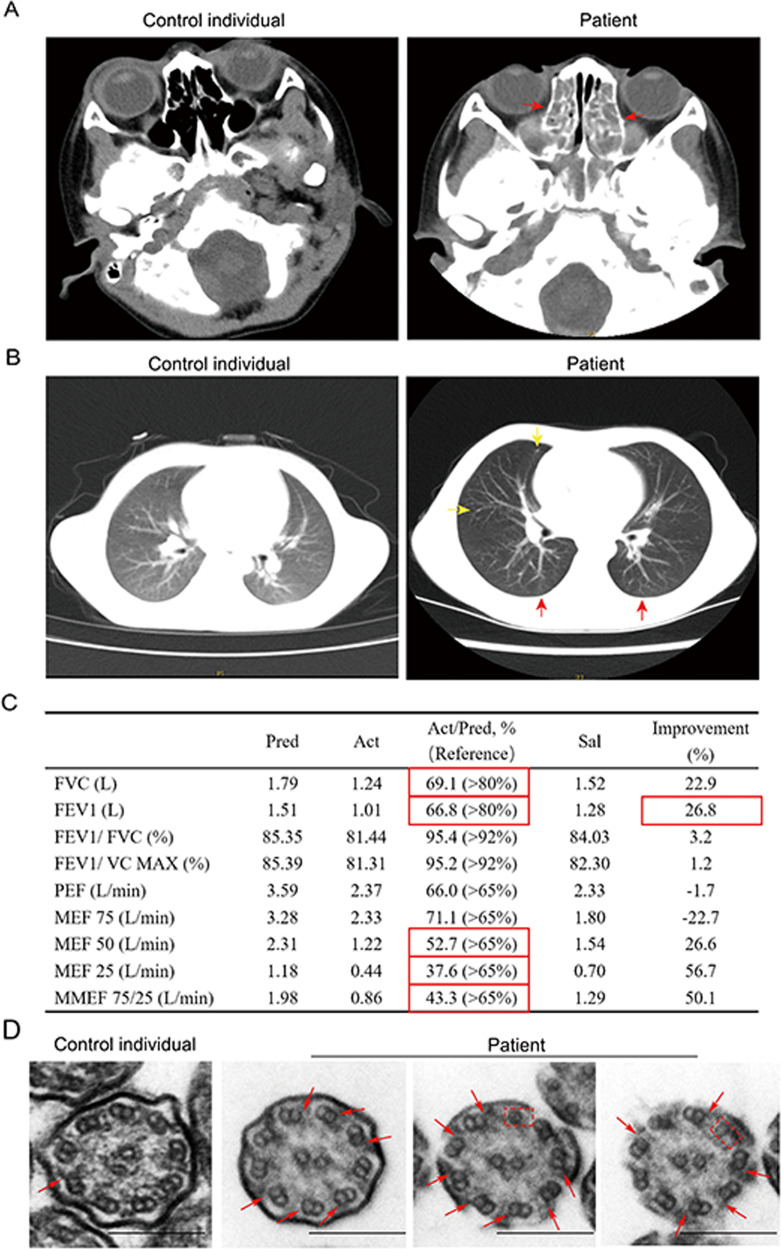
The patient with PCD shows reduced lung function and abnormal “9 + 2” structure. **A** CT scan analysis of the nasal sinuses shows pansinusitis in the patient. The red arrow reflects nasosinusitis. **B** Chest plain image of the patient by CT scan showing the bronchiectasis in the patient. The red arrow reflects pulmonary interstitial changes; the yellow arrow reflects small nodules. **C** The results of spirometry in the patient with PCD demonstrating inspiratory airflow limitation and compromised lung function. Pred: predicted value; Act: actual measured value; Act/Pred: ratio of actual measured to predicted values; Sal: measured value after inhaling salbutamol; Improvement: ratio of Sal/Pred to Act/Pred; FVC: forced vital capacity; FEV1: forced expiratory volume in one second; FEV1/FVC: ratio of FEV1 to FVC; FEV1/VC MAX: ratio of FEV1 to VC MAX; PEF: peak expiratory flow rate; MEF 75: maximal expiratory flow after 75% of the FVC has not been exhaled; MEF 50: maximal expiratory flow after 50% of the FVC has not been exhaled; MEF 25: maximal expiratory flow after 25% of the FVC has not been exhaled; MMEF 75/25: maximal mid-expiratory flow. The measurement of FVC and FEV1 are 69.1% and 66.8%, respectively, suggesting restricted ventilation dysfunction. The MEF 50 and 25 were less than 65% suggesting small airway airflow obstruction. After inhaling salbutamol, the FEV1 improved to 26.8%, suggesting positive bronchial diastolic test. Reference range is shown in bracket; red boxes indicate abnormal value. **D** Ultrastructure of the ciliary axonemes from normal child and the patient by TEM showing the ODAs defects of ciliary axonemes from the patient. The red arrows indicate the ODA structure in control individual and the lack of ODA in the patient. The red dash box indicates single microtube existing in the patient. Scale bar = 200 nm.

Low-dose computed tomography (CT) was used to visualize the nasal sinus and lungs of the patient. The results confirmed nasosinusitis and bronchiectasis in the patient (Fig. 2A, B). Furthermore, spirometry demonstrated that the patient had inspiratory airflow limitation, manifesting as low forced vital capacity (FVC) and significant bronchodilator responsiveness (Fig. 2C). TEM of the patient’s nasal biopsy showed that some of the ODA structures in the motile cilia were abolished (Fig. 2D). These results demonstrate that *DNAH9* mutations in the patient with PCD are responsible for the disease characteristics.

### *Dnah9* KD mice display typical PCD phenotypes consistent with ODA defects

**Fig. 3:**
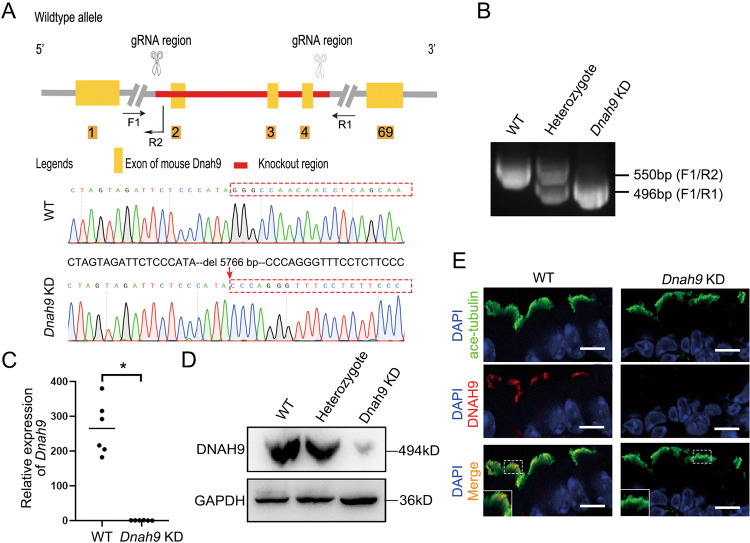
Generation and verification of *Dnah9* KD mice model. **A** The construction schematic drawing of the *Dnah9* KD model. The gRNAs target in exon2-exon5 of *Dnah9* to delete the gene region. The sanger sequence of WT and KD mice are shown; red dash box indicate different genome sequence which is attributed to deletion of 5766 bp. **B** The genotype of *Dnah9* KD, heterozygote, and WT. A single 550 bp band refers to WT, combination of 550 bp and 496 bp bands refer to the heterozygote, and single 496 bp refers to *Dnah9* KD. **C** RT-PCR of mRNA isolated from WT and *Dnah9* KD mice lung showing reduced *Dnah9* mRNA expression in the *Dnah9* KD mice. *P < 0.05. *n* = 3 per group **D** Western blot analysis is used to detect KD efficiency. **E** The immunostainings of tracheal tissue with anti-DNAH9 and anti-ace-tubulin depicting the absence of DNAH9 protein and the cluttered arrangement of cilia in *Dnah9* KD mice. Green, ace-tubulin; red, DNAH9; bule, DAPI. Scale bar = 10 µm.

To determine whether *DNAH9* deletion leads to cilium function in vivo, we generated a *Dnah9* KD mouse model using CRISPR-Cas9 (Fig. 3A). The genotypes of the mice were confirmed by PCR analysis of genomic tail DNA (Fig. 3B). And RT-PCR showed that the *Dnah9* transcript was abolished in the lung tissues of KD mice (Fig. 3C). Western blot further showed that DNAH9 protein was largely undetectable in KD mice (Fig. 3D). Moreover, IF showed that ciliary DNAH9 staining was significantly reduced in the ciliated cells of *Dnah9* KD mice (Fig. 3E).

**Fig. 4:**
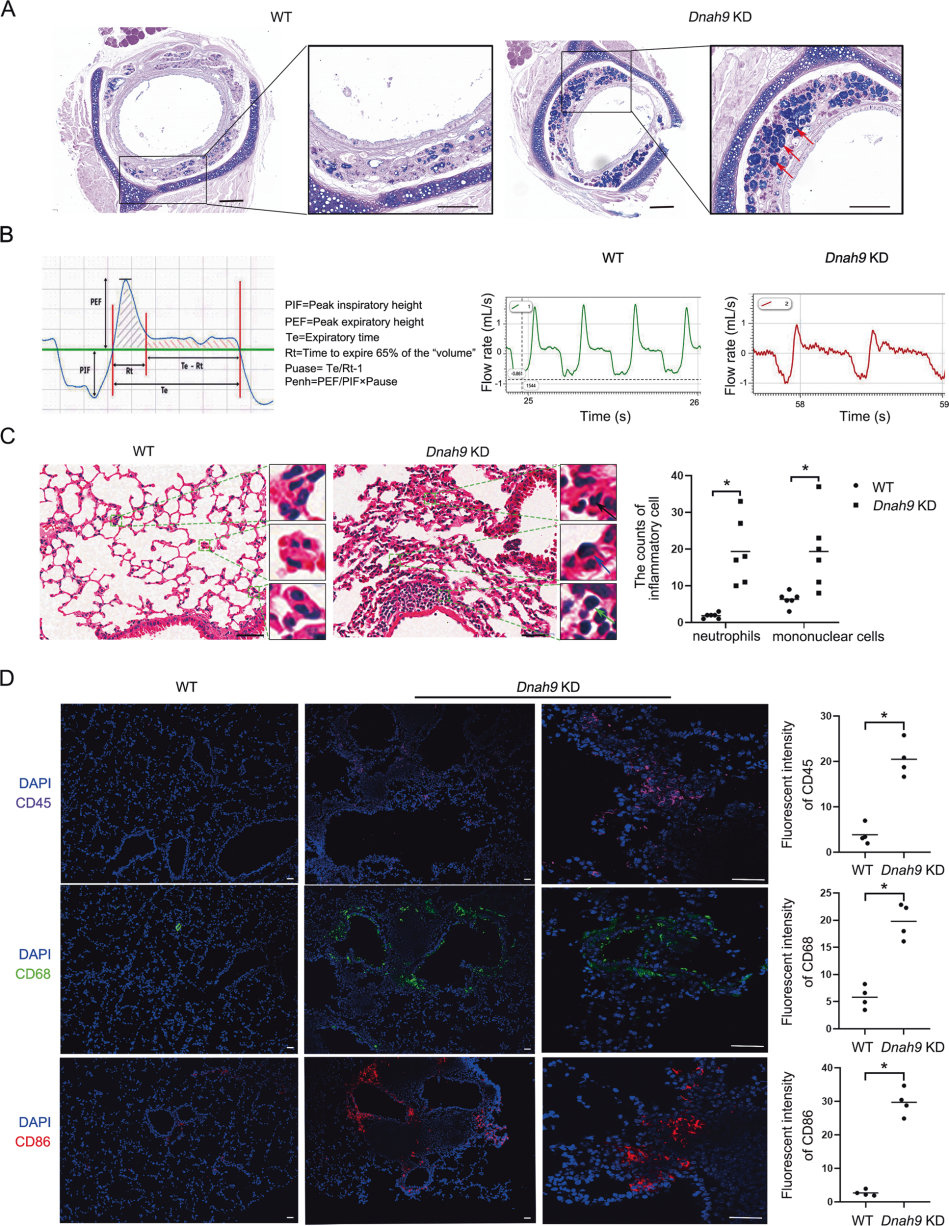
*Dnah9* KD mice show increased mucin secretion, compromised lung function, and accumulated inflammation. **A** Alcian blue/periodic acid–Schiff staining in WT and *Dnah9* KD trachea. Boxed regions are shown at higher magnification on the right. Mucus accumulation is evidently visible in the *Dnah9* KD mice (red arrows). Scale bar: left = 200 µm, right = 50 µm. **B** Breathing curve demonstrating lung function parameters of WT and *Dnah9* KD mice. The left diagram represents a unit of the WBP curve, including an inspiratory and expiratory process each. **C** H&E staining of lung sections in WT and *Dnah9* KD mice at 40 days old. Compared to the WT mice, the lung of *Dnah9* KD mice shows increased infiltration of inflammatory cells. Mononuclear cells (including lymphocytes and macrophages) and neutrophils are significantly increased, as depicted in the scatter plot. The green dotted boxes indicate the magnified region of the lung slice. The green, black, and blue arrows represent lymphocytes, neutrophils, and macrophages, respectively. Equal-sized 5% area of the sections are chosen for counting. The statistical results are shown in the right panel. Scale bar = 20 μm. *P < 0.05, *n* = 6 per group. **D** The immunostaining of immunocytes (CD45: leucocyte marker; CD68: macrophage marker; CD86: monocyte as well as T and B lymphocyte marker), showing increased positive signals in lung section of *Dnah9* KD mice model. Purple, CD45; green, CD65; red, CD86; blue, DAPI. Scale bar = 50 μm. *P < 0.05, *n* = 3 per group.

Mucin clearance is the most crucial function of airway motile cilia. Intriguingly, through combined Alcian blue-periodic acid-Schiff (AB-PAS) staining, we found a significant increase of acidic mucin-secreting mucosal glands in *Dnah9* KD mice compared to that in control mice (Fig. 4A). Mouse pulmonary function was determined by following parameters, including inspiratory-to-expiratory time (Ti/Te), PEF, PIF, Rpef, Mv, Volbal, PenH, and EF50. The results showed that except PIF, the above parameters were significantly reduced in the *Dnah9* KD mice, indicating increasing airway obstruction and worsening bronchoconstriction (Fig. 4B; Supplementary Fig. 1). Notably, *Dnah9* KD mice displayed a slight increase in PIF, suggesting that the inspiratory muscles were unaffected by *DNAH9* depletion (Fig. 4B; Supplementary Fig. 1). Hematoxylin and eosin (H&E) staining demonstrated an increased number of neutrophils and mononuclear cells, including lymphocytes and macrophages, in the lungs of *Dnah9* KD mice (Fig. 4C). Fluorescence intensity of inflammatory cell markers, including leukocytes, macrophages, monocytes, and T and B lymphocytes, was significantly enhanced in the lungs of *Dnah9* KD mice, reflecting severe infections in the respiratory tract of *Dnah9* KD mice (Fig. 4D).

Considering that one of the major clinical phenotypes of PCD is male infertility, we investigated whether spermatogenesis was affected and characterized sperm parameters in the KD mice. We investigated the motility of sperm isolated from cauda epididymis using computer-assisted sperm analysis (CASA) and found that motility parameters were reduced in KD mice (Supplementary Fig. 2A). We further observed that the ultrastructure of *Dnah9* KD mouse spermatozoa showed no apparent difference compared to that in the WT mice by TEM analysis (Supplementary Fig. 2B). However, obvious coiled flagella in partial *Dnah9* KD spermatozoa were noted (Supplementary Fig. 2C). In addition, DNAH9 was detected in the cytoplasm of spermatogonia and early and late spermatids and gradually gathered in the flagella during spermatogenesis (Supplementary Fig. 2D). Notably, *Dnah9* showed the highest expression in lung tissue and relatively high expression in the brain and testis (Supplementary Fig. 2E). The *Dnah9* expression exhibited relative stability during the different developmental stages of spermatogenesis (Supplementary Fig. 2F). Although sperm cells showed partially abnormal flagella development and reduced motility, *Dnah9* KD mice were normally fertile (data not shown), suggesting that *DNAH9* is dispensable for mouse fertility.

**Fig. 5:**
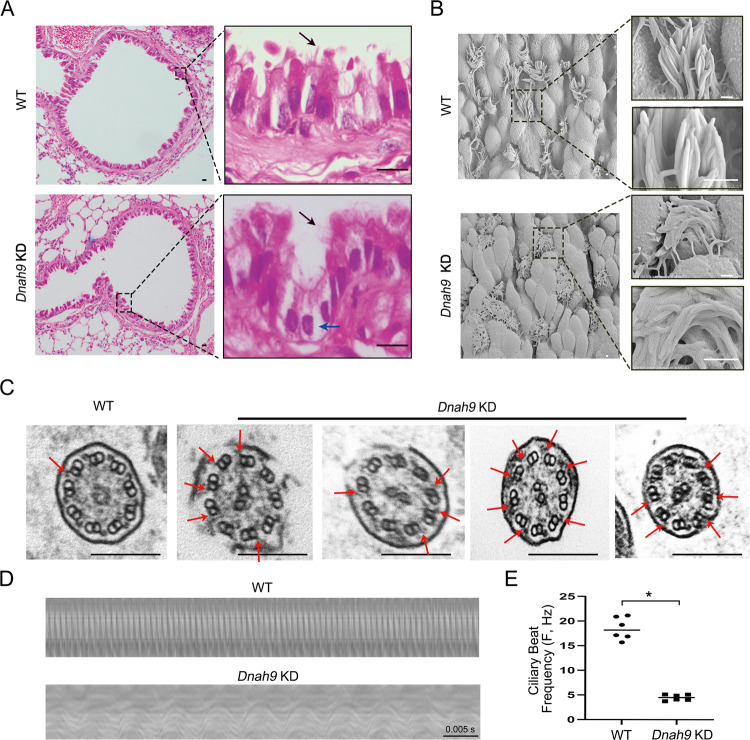
Abnormal cilia structure and function in *Dnah9* KD mice. **A** H&E staining of bronchi in lung sections in WT and *Dnah9* KD mice at 40 days old. Compared to the WT, the tracheal epithelia of *Dnah9* KD mice show vacuolization (blue arrows), and the distribution of cilia seems more clustered (black arrows). Boxed regions on the right depict higher magnifications. Scale bar = 10 μm. **B** The SEM showing surface of tracheal epithelia from WT and *Dnah9* KD mice. Boxed regions are shown at higher magnification on the right. Scale bar = 9 μm. **C** TEM images of tracheal epithelium cilia for WT and *Dnah9* KD mice. The axonemal structure is regular but shows a partially distal absence of ODAs in tracheal epithelium cilia of *Dnah9* KD mice (red arrow). Scale bar = 200 nm. **D** Representative kymograph of 40-day-old WT and *Dnah9* KD nasal cilia from high-speed video microscopy study. Scale bar = 0.005 s. **E** The CBF of *Dnah9* KD mice is significantly reduced compared with WT mice. *P < 0.05. *n* = 6 per group.

To examine whether the phenotypes of the airway motile cilia were affected, we performed H&E staining. We found that the multiciliated airway epithelial structure in *Dnah9* KD mice was severely vacuolated, and the cilia appeared more crooked (Fig. 5A). SEM was used to visualize cilia morphology. Compared to the cilia of the WT mice which showed a straight and uniform direction protruding from the cell plasma membrane, the cilia from *Dnah9* KD were curved, non-directional, and crawled outside the cells (Fig. 5B). In addition, the distal regions of motile cilia were substantially curved, which coincided with the distal localization of DNAH9 in motile cilia (Fig. 5B). Additionally, TEM analysis showed that the ODA ultrastructure was absent in the distal cilia region (Fig. 5C), indicating that the KD mice phenocopied the patients with PCD. Furthermore, high-speed video microscopy showed that the CBF of the KD mice was significantly reduced compared to that of the WT mice (Fig. 5D, E; Supplementary Movie 1–2), confirming that *DNAH9* deletion impairs ciliary motility.

### DNAH9 interacts with GAS8 and CCDC114 and affects their protein expression in cilium

**Fig. 6:**
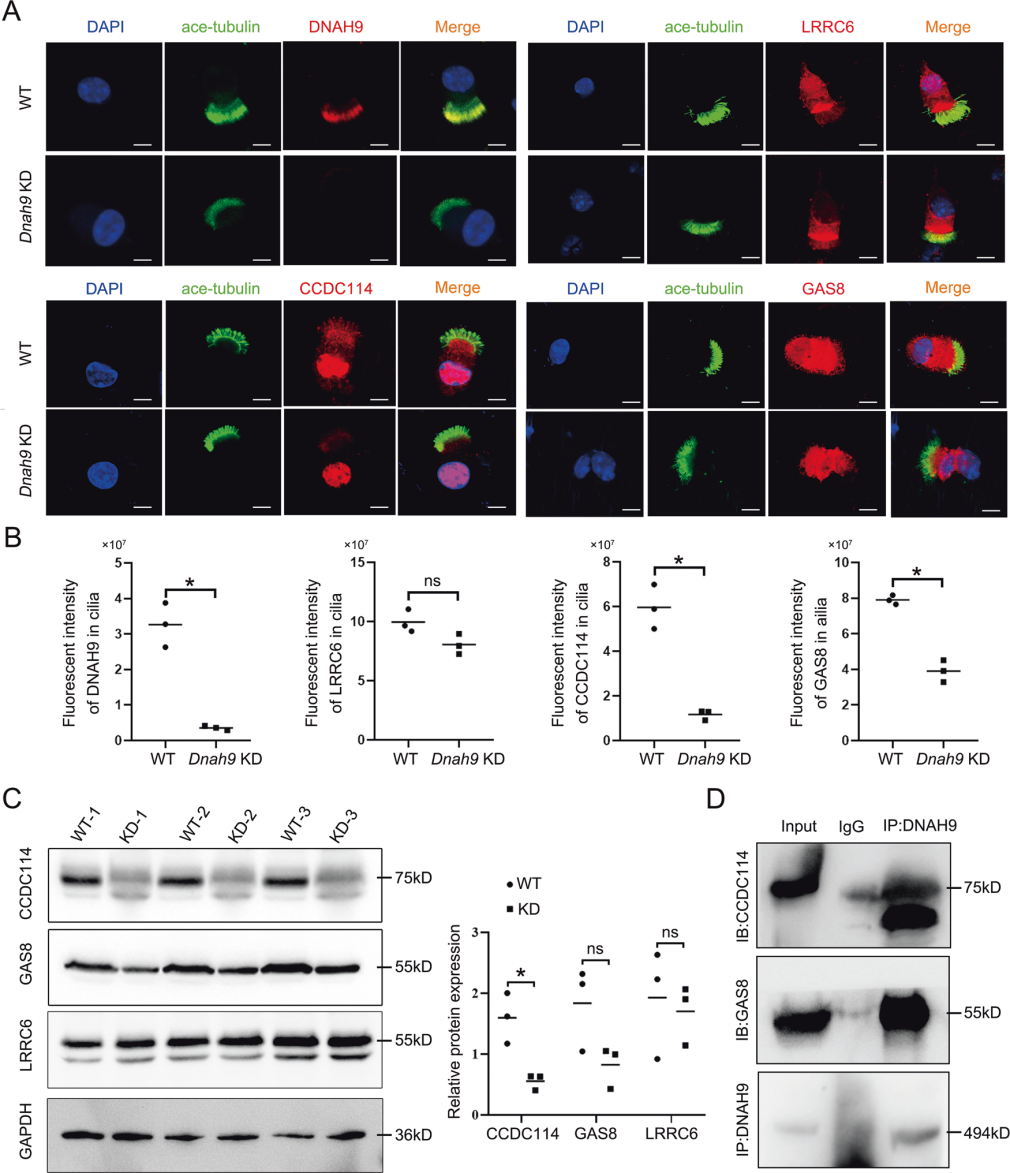
Ciliated cells from *Dnah9* KD mice show reduced expression of interactors CCDC114 and GAS8 protein. **A** Immunostaining analysis of DNAH9, CCDC114, LLRC6, and GAS8 in ciliated cells from *Dnah9* KD nasal epithelium. Green, ace-tubulin; red, DNAH9, LRRC6, CCDC114, and GAS8; blue, DAPI; Scale bar = 10 µm. **B** Results of immunofluorescence images quantification corresponding to ciliated cells in (**A**). *P < 0.05; ns no significance, *n* = 3 per group. **C** Western blot analysis showing the protein expression of CCDC114, GAS8, and LRRC6 in lung tissues of WT and *Dnah9* KD mice. GAPDH is the endogenous control. The right pattern is the statistical analysis of relative protein intensities revealed by western blot. *P < 0.05; ns no significance, *n* = 3 per group. **D** Co-IP results demonstrating interactions between DNAH9 and CCDC114/GAS8.

To further investigate whether the absence of DNAH9 affects ODA-associated proteins, we performed immunostaining to examine markers of the ODA-DC, dynein arm assembly, and nexin-dynein regulatory complex (N-DRC) in multiciliate cells from nasal tissue. Notably, our immunostaining results showed the expression of GAS8 and CCDC114 in cilia was dramatically decreased upon deletion of *DNAH9* (Fig. 6A, B), indicating a structural impairment in the ODA-DC and N-DRC in *Dnah9* KD mice. In contrast, dynein axonemal assembly factor 11 (LRRC6) was not obviously affected. Furthermore, western blot analysis showed that the expression of CCDC114 was significantly reduced in KD mice, and GAS8 expression also showed a decreasing trend, though no significant differences were found between WT and KD mice (Fig. 6C). Co-IP assays revealed that both CCDC114 and GAS8 interacted with DNAH9 (Fig. 6D). Our results collectively demonstrate that DNAH9 implements the ODA function, possibly by cooperating with CCDC114 and GAS8.

### The airway organoid model from *Dnah9* KD mice can phenocopy the patient with PCD

**Fig. 7:**
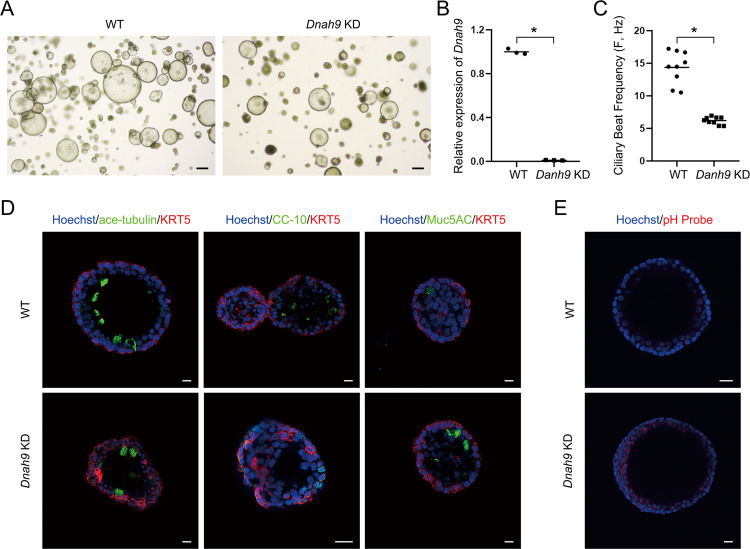
*Dnah9* KD mouse organoid shows reduced ciliary beating and increased secretion of inflammatory factors. **A** Bright-field images depicting mouse airway organoid phenotypes. Scale bar **=** 100 µm. **B** RT-PCR shows low DNAH9 expression in *Dnah9* KD mouse airway organoid relative to WT. *P < 0.05; *n* = 3 per group. **C** CBF of *Dnah9* KD group was significantly lower than WT group. *P < 0.05. *n* = 10 per group. **D** Whole-mount mouse airway organoid immunofluorescence shows markers for basal cells (KRT5, red), ciliated cells (acetylated a-tubulin, green), club cells (CC-10, green) and goblet cells (MUC5AC, green). Scale bar **=** 10 µm. **E** Confocal microscopy images of mouse airway organoid with a pH probe show lower pH value in the *Dnah9* KD group than WT. The stronger red color indicates lower pH value in the epithelial cell of the *Dnah9* KD group. Scale bar **=** 10 µm.

**Fig. 8:**
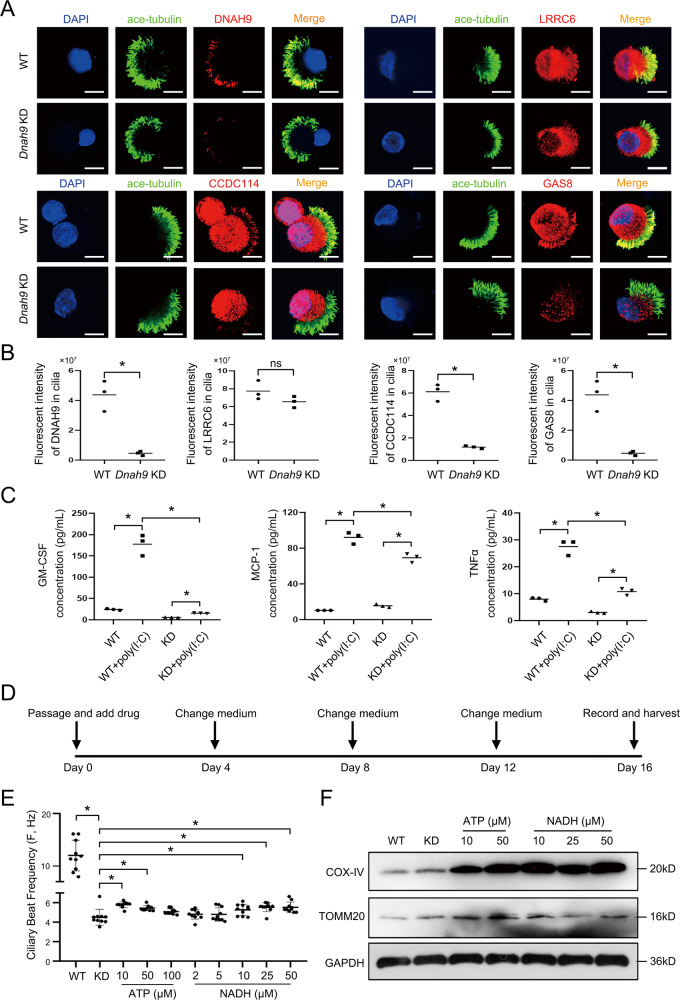
Drugs targeting mitochondrial could rescue the cilia beating and related phenotypes. **A** Expression analysis of DNAH9, CCDC114, LLRC6, and GAS8 in ciliated epithelium digested from WT and *Dnah9* KD mouse airway organoid. Green, ace-tubulin; red, DNAH9, LRRC6, CCDC114, and GAS8; blue, DAPI. Scale bar **=** 10 µm. **B** The quantified DNAH9, LRRC6, CCDC114, and GAS8 fluorescent intensities in cilia analyzed by ImageJ in (**A**). *P < 0.05; ns no significance, *n* = 3 per group. **C** The expression of immune factors in mouse airway organoid after 4-day poly (I:C) treatment. *P < 0.05; *n* = 3 per group. **D** Stepwise protocol for the organoid drug screen. **E** CBF of *Dnah9* KD mouse airway organoid after 16-day drug treatment. *P < 0.05, *n* = 10 per group. **F** Western blot of TOMM20 and COX-IV in KD mouse airway organoid treated with ATP (10 µM, 50 µM) and NADH (10 µM, 25 µM, 50 µM) and WT organoid.

Epithelial cells from WT and *Dnah9* KD mice were observed to form hollow spherical tracheas, which is a typical morphology of trachea organoids (Fig. 7A). Consistent with the results mentioned earlier, the expression of *Dnah9* in the *Dnah9* KD organoid was significantly reduced compared to that in the WT organoid (Fig. 7B). To further investigate the function of motile cilia in organoids, CBF was recorded using a high-speed video microscope. Similarly, we found that the CBF of motile cilia in the *Dnah9* KD organoid was significantly lower than that in the WT organoid (Fig. 7C; Supplementary Movie 3–4). Based on the immunostaining results of different cell markers in the organoids, airway organoids retained the main original cell types in the tracheal tissue. Basal cells were located on the basal side; ciliated cells, club cells, and goblet cells were luminally localized, staining with KRT5, acetylated α-tubulin, CC-10, and Muc5AC, respectively. Moreover, we observed a bigger proportion of goblet cells in KD mouse organoid than that in WT, whereas other cell types showed no significant changes (Fig. 7D). Considering the critical role of pH value on mucin secretion function and the secretory function of goblet cells [34], the pH value was tested with a previously demonstrated pH probe [35], varying dramatically within the appropriate pH range (pH value 4.5–8.0). In the acidic cell environment, the pH probe would emit red fluorescence; the stronger acidic condition of cell is, the higher intensity of red fluorescence will be detected. Our results revealed that probe staining of the *Dnah9* KD organoid was stronger than that in the WT organoid, implying the pH value of the *Dnah9* KD organoid was much lower than that of the WT organoid, which is in accordance with previous AB-PAS staining results (Fig. 7E). Moreover, we examined the expression of DNAH9 and ODA-associated proteins (CCDC114, GAS8, LRRC6) in ciliated cells from mouse airway organoids using IF (Fig. 8A, B). The results were consistent with the staining of multiciliated cells from nasal tissue, which showed almost undetectable DNAH9, diminished CCDC114 and GAS8 expression, and an equivalent level of LRRC6 in *Dnah9* KD organoids compared with that in the WT. Therefore, the mouse airway organoid model can recapitulate the complexities of airway structure and maintain airway features in genotype-phenotype correlation and cellular function after long-term expansion in vitro.

One of the major advantages of organoids for PCD research is that organoids can be used to examine the immune response [36]. We detected cytokines after poly (I:C) treatment in WT and *Dnah9* KD organoids, and the results demonstrated that GM-CSF, MCP-1, and TNFα levels were significantly increased in poly (I:C) treatment groups of WT and *Dnah9* KD organoids, compared to untreated groups, while the rising level of *Dnah9* KD organoids was far less than that in WT organoids (Fig. 8C). In addition, other cytokines including interleukin 1 alpha (IL-1α), keratinocyte chemoattractant (KC), macrophage inflammatory protein-2 (MIP-2), and C-X-C motif chemokine 5 (LIX) showed similar tendency (Supplementary Fig. 3), indicating that *DNAH9* depletion reduced cytokine release in the organoid system.

### Drugs targeting mitochondrial NADH can improve cilia motility in *Dnah9* KD organoids

To evaluate *Dnah9* KD airway organoids as a functional PCD disease model, we tested whether organoids could be suitable for drug sensitivity screening. ATP and NADH were selected to treat *Dnah9* KD airway organoids with at least three concentrations per drug. After a 16-day treatment, the CBF of organoids was recorded, and the samples were harvested (Fig. 8D). We observed that several treatments improved the CBF of the KD organoids. The CBF in samples treated with ATP 10 µM (5.793 ± 0.09344 Hz, *n* = 10), 50 µM (5.437 ± 0.08295 Hz, *n* = 10), NADH 10 µM (5.269 ± 0.1804 Hz, *n* = 10), 25 µM (5.532 ± 0.1457 Hz, *n* = 10), and 50 µM (5.523 ± 0.1720 Hz, *n* = 10) was significantly higher than that of the KD control sample (4.499 ± 0.2601 Hz, *n* = 10) (Fig. 8E). Furthermore, western blot results showed that the cytochrome c oxidase-Cox-IV component was significantly increased after NADH treatment (Fig. 8F), supporting the notion that the rescue effects of cilia beating are related to the improvement of mitochondrial function. TOMM20, a mitochondrial outer membrane structural protein, was not significantly affected in *Dnah9* KD mice.

## Discussion

In the current study, we identified a novel *DNAH9* mutation in a patient with PCD and conducted bioinformatics and expression analysis to show that the damaging compound mutation of *DNAH9* leads to PCD phenotypes associated with cilia defects. We constructed *Dnah9* KD mice and airway organoids to serve as disease models and drug screening platforms. In addition, we identified mitochondria-targeting drugs that can restore the reduced CBF, at least partially, in *Dnah9* KD airway organoids.

DNAH9 is an ODA2 type dynein heavy chain protein; recent studies have shown that it is precisely expressed in the distal region of cilia, and mutations have been described in patients with PCD who have inverse or productive cough [5, 11, 37] [38]. Therefore, an in vivo model is urgently required to understand the pathophysiology of PCD associated with *DNAH9* mutation. The KD mouse model showed abnormal cilial morphology, slower cilia wave, compromised lung function, and an increased percentage of immune cells in the bronchi of the lung. The above results indicated that the *Dnah9* KD mouse model is a perfect match for the symptoms of patients with PCD harboring *DNAH9* mutations, and further confirmed the function of DNAH9 in cilia motility.

Investigating how the *DNAH9* mutation led to dysfunctional ciliary function, we found an interaction between DNAH9 and CCDC114/GAS8, indicating that the absence of DNAH9 could affect the expression of CCDC114 and GAS8. CCDC114 is a component of the ODA docking complex, and GAS8 is a structural protein of the N-DRC complex, which is adjacent to the ODA complex [39, 40]. Loss of *DNAH9* function affected the expression of ODA-associated proteins. Overexpression of *Ccdc*114 cannot rescue cilia beating defects in *Dnah9* KD organoids, indicating that other proteins are also required for the normal expression of DNAH9 (data not shown). Whether the phenotypes are directly related to the reduced interaction of ODA-DC and N-DRC needs further investigations [1, 41]. Nevertheless, our KD mouse model represents the key symptoms of patients with PCD.

The PCD organoid developed in this study represents an exciting in vitro model superior to in vivo models in several respects. Unlike primary lung cilia culture, organoids represent a more physiologically relevant model that is a less expensive and time-consuming form of mouse breeding [36, 42]. Lung ciliated cell culture has been traditionally used to investigate the mechanism of the PCD phenotypes [41, 43]. However, cilial defects can reportedly affect ciliary growth and other cells, such as goblet cells [44]. Goblet cells are major secretory cells in the superficial epithelium of the trachea-bronchial airway, and their function is closely related to ciliary function. For example, an antibody blocking the Notch pathway could induce the transdifferentiation of goblet cells into ciliated cells [45]. In our model, we observed that *Dnah9* KD could cause an over-population of goblet cells, whereas other cell types showed no significant changes. Therefore, the increase in goblet cell numbers could be due to compensatory effects. Using a pH-sensitive dye that we developed previously [35], we found that the intracellular pH value was significantly decreased in the epithelial cells of the *Dnah9* KD organoid model. Since pH is a critical factor determining mucin clearance [34], the cilial cell function defects of *Dnah9* KD mice could also affect mucin secretion through goblet cell overproliferation. Additionally, the immune cytokine profile indicated that the levels of some specific cytokines, including GM-CSF, IL-1α, and TNFα, were significantly decreased. Notably, GM-CSF is a critical marker in severe COVID-19 infection in the lung [46], indicating that some specific cytokines, such as GM-CSF, could serve as critical cytokine mediators for lung lesions caused by *DNAH9* mutations and COVID-19 infection. Thus, immune therapy deserves further exploration to examine the possible effects of PCD [44]. In short, the organoid model from *Dnah9* KD mice provides a valuable tool for further clarifying the functions of the ODA protein in PCD, other cilia-defective disease models, and drug screening.

Although PCD is mainly caused by dynein arm mutations, an increasing number of studies have shown that other alterations, such as energy abnormalities, could also be responsible for ciliary growth defects [26], and the mitochondrial pathway represents a potential therapeutic target to restore normal ciliary function [44, 47]. Therefore, a drug-screening model was established to identify novel treatments for PCD. To the best of our knowledge, this is the first drug-screening organoid model developed for the ciliated cell model of ODA defects. We chose several drugs targeting the mitochondria based on a recent study that proved mitochondrial dysfunction is closely related to ciliary function [26]. Our results show that NADH represents a viable drug that can at least partially restore ciliary motility. Therefore, our results demonstrate that the organoid model could provide a platform to further screen more potent drugs for PCD therapy.

The patient-derived PCD organoid model represents an excellent opportunity to understand the pathophysiology of PCD, as organoids can be developed with patient-specific biopsy samples. In the clinic, ODA genes such as *DNAH5* are more commonly mutated in PCD, and their mutations have caused variable phenotypes based on different mutation sites [37, 48]. This shows that individual patient-specific PCD organoids could provide a personalized model to understand the correlation of mutation profiling with the phenotypes, as well as explore effective intervention drugs [48–50]. In this regard, our drug screening results showed that the proof-of-concept tracheal organoid developed in this study could be used as an excellent PCD disease model platform using tissues from either KD mice or PCD patients and that precise medicine-based drug screening in patients with PCD is possible [51].

## Supplementary information

Supplementary Fig. 1. Lung function parameters demonstrating airway obstruction and chronic bronchitis in Dnah9 KD mice

Supplementary Fig. 2. DNAH9 is dispensable for mouse fertility and its expression pattern in the mouse.

Supplementary Fig. 3. The expression of other immune factors in mouse airway organoid after 4-day poly(I:C) treatment.

Supplemental table 1. Analysis of variant in DNAH9 for the patient with PCD

Supplementary Movie 1 Ex-vivo video-microscopy recording of nasal mucosal cilia in the P40 wildtype.

Supplementary Movie 2 Ex-vivo video-microscopy recording of the nasal mucosal cilia in the P40 Dnah9 KO mice.

Supplementary Movie 3 Ex-vivo video-microscopy recording of wildtype mouse airway organoid.

Supplementary Movie 4 Ex-vivo video-microscopy recording of Dnah9 KO mouse airway organoid.

Supplementary legends

Western blot raw data

Reproducibility checklist
