# Supplementary figures and images for: Dnah9 mutant mice and organoid models recapitulate the clinical features of patients with PCD and provide an excellent platform for drug screening
Source: Cell Death Dis. 2022 Jun 21;13(6):559. doi: 10.1038/s41419-022-05010-5 (PMC9210797; doi:10.1038/s41419-022-05010-5)

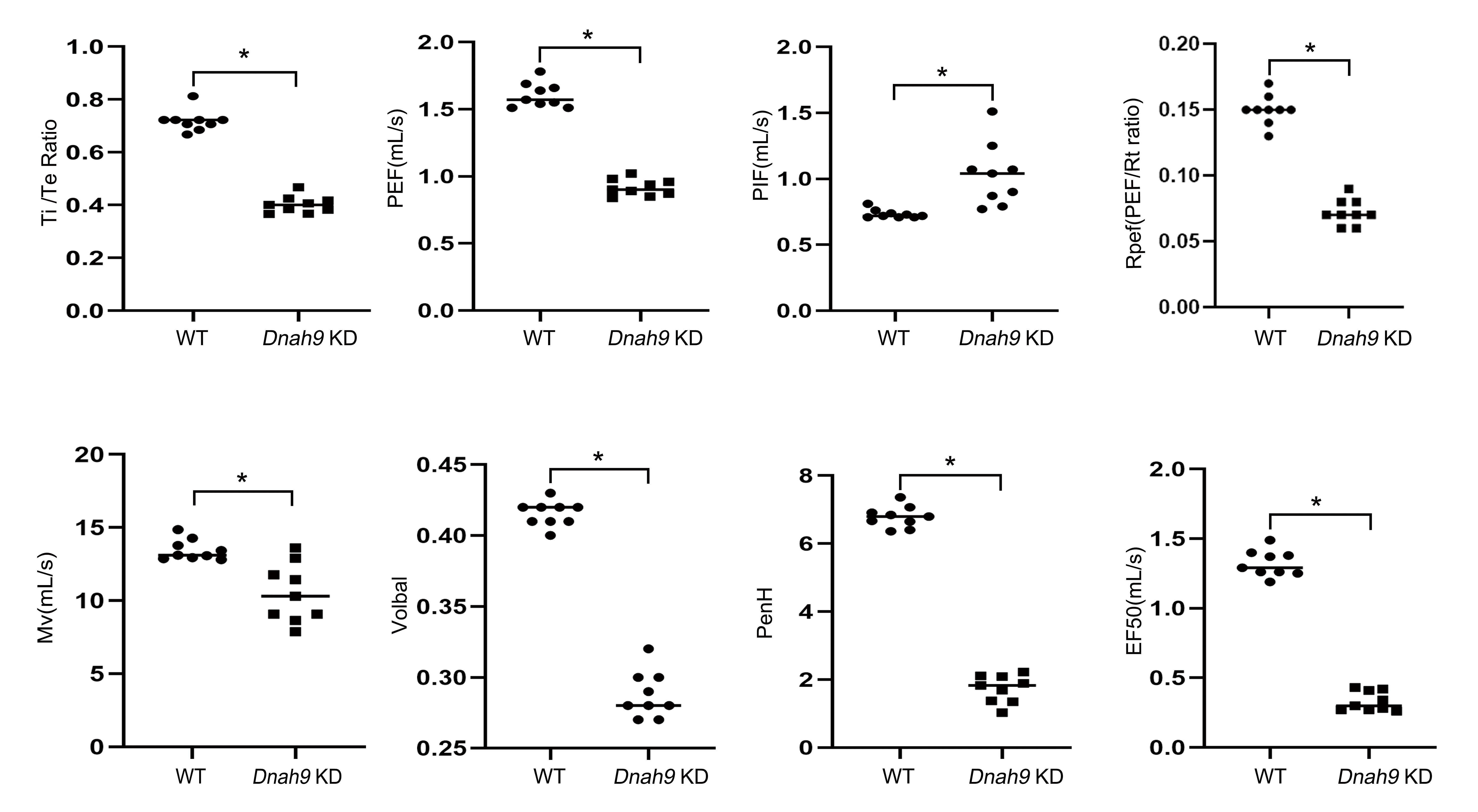

Supplement: Supplementary file 1 — Supplementary Fig. 1. Lung function parameters demonstrating airway obstruction and chronic bronchitis in Dnah9 KD mice [file 41419_2022_5010_MOESM1_ESM.png]

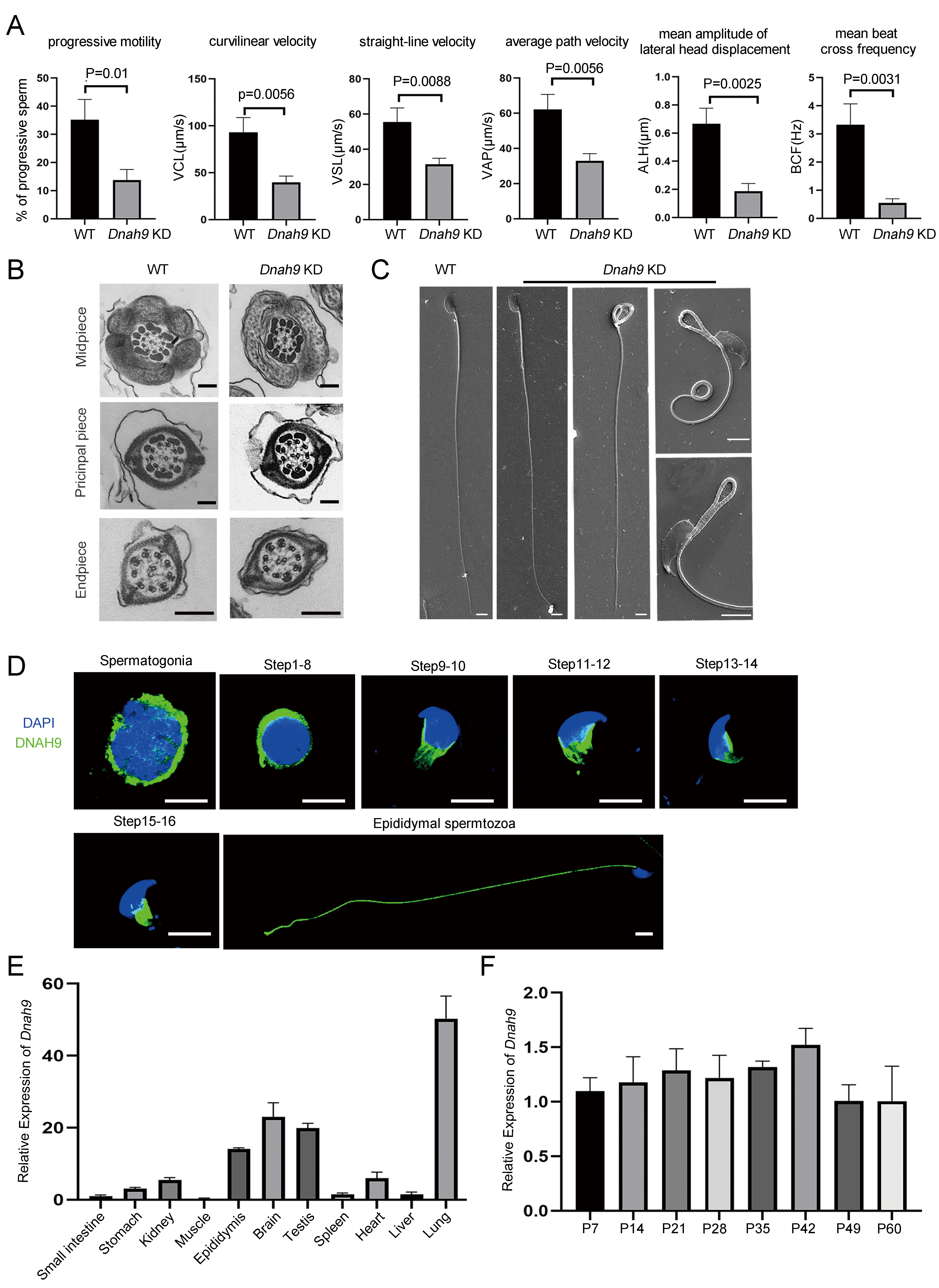

Supplement: Supplementary file 2 — Supplementary Fig. 2. DNAH9 is dispensable for mouse fertility and its expression pattern in the mouse. [file 41419_2022_5010_MOESM2_ESM.png]

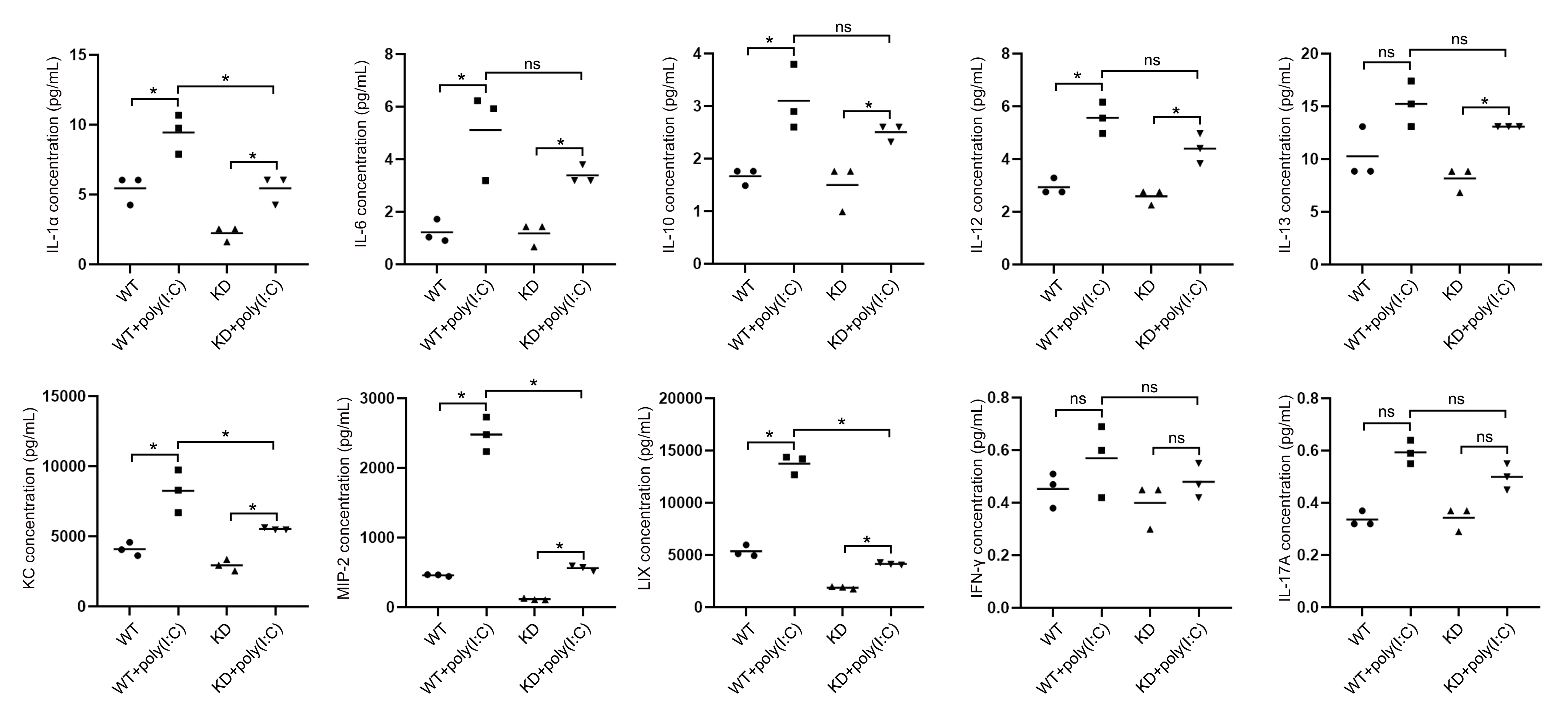

Supplement: Supplementary file 3 — Supplementary Fig. 3. The expression of other immune factors in mouse airway organoid after 4-day poly(I:C) treatment. [file 41419_2022_5010_MOESM3_ESM.png]
