# Supplementary material for: Dnah9 mutant mice and organoid models recapitulate the clinical features of patients with PCD and provide an excellent platform for drug screening
Source: Cell Death Dis. 2022 Jun 21;13(6):559. doi: 10.1038/s41419-022-05010-5 (PMC9210797; doi:10.1038/s41419-022-05010-5)
Supplement: Supplementary file 4 — Supplemental table 1. Analysis of variant in DNAH9 for the patient with PCD [file 41419_2022_5010_MOESM4_ESM.docx]

**Supplemental table 1 variant analysis of patient harboring *DNAH9* mutations**

| Genomic mutation | Protein changes | Mutation type | Genotype | Allele frequency in ExAC | 1000 Genomes Project | GnomAD | SIFT | Polyphen-2 | Mutation Taster |
| --- | --- | --- | --- | --- | --- | --- | --- | --- | --- |
| c.6431G>A | p. R2144Q | Missense | Heterozygous | 0 | 0 | 1.63E-05 | D | D | D |
| c.12835G>A | p. G4279S | Missense | Heterozygous | 0.0005 | 0.00379 | 0.0009 | T | D | D |

a: D means deleterious, T means tolerate in SIFT, b: D means probably damaging in Polyphen-2, c: D means disease causing in Mutation Taster.
