# Supplementary material for: Dnah9 mutant mice and organoid models recapitulate the clinical features of patients with PCD and provide an excellent platform for drug screening
Source: Cell Death Dis. 2022 Jun 21;13(6):559. doi: 10.1038/s41419-022-05010-5 (PMC9210797; doi:10.1038/s41419-022-05010-5)
Supplement: Supplementary file 9 — Supplementary legends [file 41419_2022_5010_MOESM9_ESM.docx]

**Supplementary Fig. 1. Lung function parameters demonstrating airway obstruction and chronic bronchitis in *Dnah9* KD mice.** Ti/Te: The ratio of inspiratory time to expiration time, indicating airway obstruction, Te: elongation indicated chronic bronchitis in *Dnah9* KD mice; PEF**:** peak expiratory flow rate (mL/s)**;** PIF: peak inspiratory flow rate (mL/s), showing inspiratory muscle strength; Rt: time to expire 65% of the “volume”; Rpef: the ratio of time to PEF to Rt, indicating respiratory muscle strength and small airway obstruction; Mv: minute ventilation (mL); Volbal: respiration ratio; Penh: quantify the degree of bronchoconstriction; EF50: expiratory flow when exhaling 50% air (mL/s), an indicator of obstructive ventilatory impairment. *P <0.05; n=9 per group.

**Supplementary Fig. 2. DNAH9 is dispensable for mouse fertility and its expression pattern in the mouse. A.** Computer-assisted sperm analyses (CASA) comparing WT and *Dnah9* KD sperm. *Dnah9* KD sperm have reduced velocity parameters: curvilinear velocity (VCL), straight-line velocity (VSL), and average path velocity (VAP), together with reduced amplitude of lateral head displacement (ALH) and the beat-cross frequency (BCF). **B.** Transmission electron microscopy analysis for spermatozoa of WT and *Dnah9* KD mice demonstrate seemingly normal axoneme. Scale bar = 100 nm. **C.** SEM analysis of spermatozoa obtain from WT and *Dnah9* KD mice. Partial spermatozoa from *Dnah9* KD mice show bent and coiled flagella. Scale bar, 5 μm. **D.** The IF detection indicates that DNAH9 is dominantly expressed in the cytoplasm of the spermatogonia, spermatocyte, round spermatids (steps 1–8), early elongating spermatids (steps 9–12), as well as late elongating spermatids (steps 13–14) and in the flagella of epididymal spermatozoa. Green, DNAH9; blue, DAPI. Scale bar = 5 µm. **E.** The result of RT-PCR reveals the expression of *Dnah9* in different mouse tissues. Quantification of the RT-PCR results by a histogram according to the cycle threshold value. **F.** RT-PCR analysis reveal the expression of *Dnah9* in different development stages of mouse testes. Quantification of the RT-PCR results is presented with histogram according to the cycle threshold value.

**Supplementary Fig. 3.** **The expression of other immune factors in mouse airway organoid after 4-day poly(I:C) treatment.** The concentration of all immune factors is increased after poly(I:C) treatment both in WT and *Dnah9* KD organoids. IL-1α, KC, MIP-2, and LIX are significantly decreased both at basal level and after poly (I:C) treatment in *Dnah9* KD mouse airway organoid. *P < 0.05; n=3 per group.

**Supplemental table 1. Analysis of variant in *DNAH9* for the patient with PCD**

**Supplementary Movie 1. Ex-vivo video-microscopy recording of** **nasal mucosal cilia in the 40-day-old WT mice.** Nasal mucosal cilia in the WT are motile and display a normal beating pattern (video captured at a rate of 200 fps and playback rate of 5 fps); scale bar =10 μm.

**Supplementary Movie 2. Ex-vivo video-microscopy recording of the nasal mucosal cilia in the 40-day-old *Dnah9* KD mice.** Nasal mucosal cilia are slow or immotile and show rigid cilia beating patterns (video captured at a rate of 200 fps and played back at a rate of 5 fps); scale bar, 10 μm.

**Supplementary Movie 3. Ex-vivo video-microscopy recording of WT mouse airway organoid.** (Video captured at a rate of 200 fps and playback rate of 5 fps). Scale bar = 100 μm.

**Supplementary Movie 4. Ex-vivo video-microscopy recording of *Dnah9* KD mouse airway organoid.** (Video captured at a rate of 200 fps and playback rate of 5 fps). Scale bar = 100 μm.
