# Supplementary material for: Dnah9 mutant mice and organoid models recapitulate the clinical features of patients with PCD and provide an excellent platform for drug screening
Source: Cell Death Dis. 2022 Jun 21;13(6):559. doi: 10.1038/s41419-022-05010-5 (PMC9210797; doi:10.1038/s41419-022-05010-5)

Figure 1C

From left to right, the sample order is NC, WT, *DNAH9* MUT

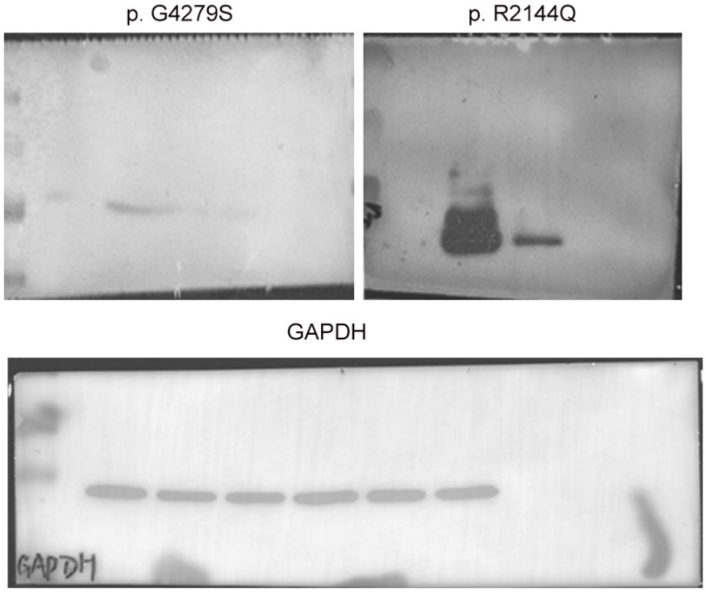

Figure 3D

From left to right, the sample order is WT, Heterozygote, *Dnah9* KD

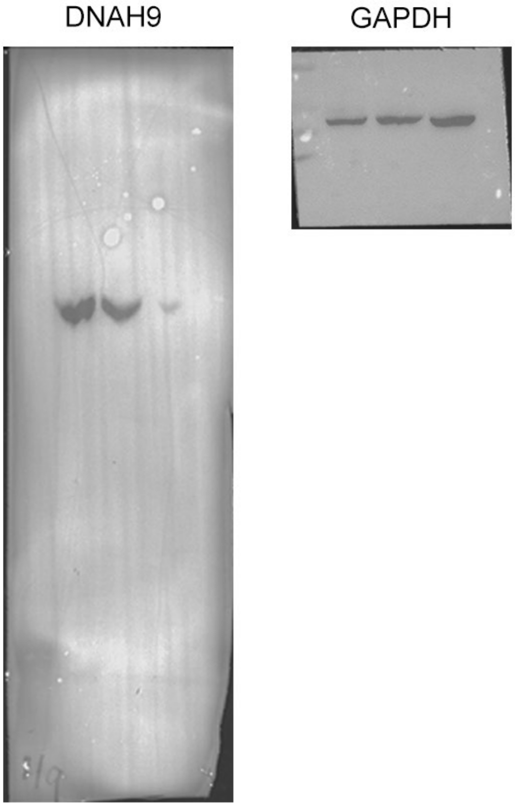

Figure 6C

From left to right, the sample order is WT, *Dnah9* KD, WT, *Dnah9* KD, WT, *Dnah9* KD

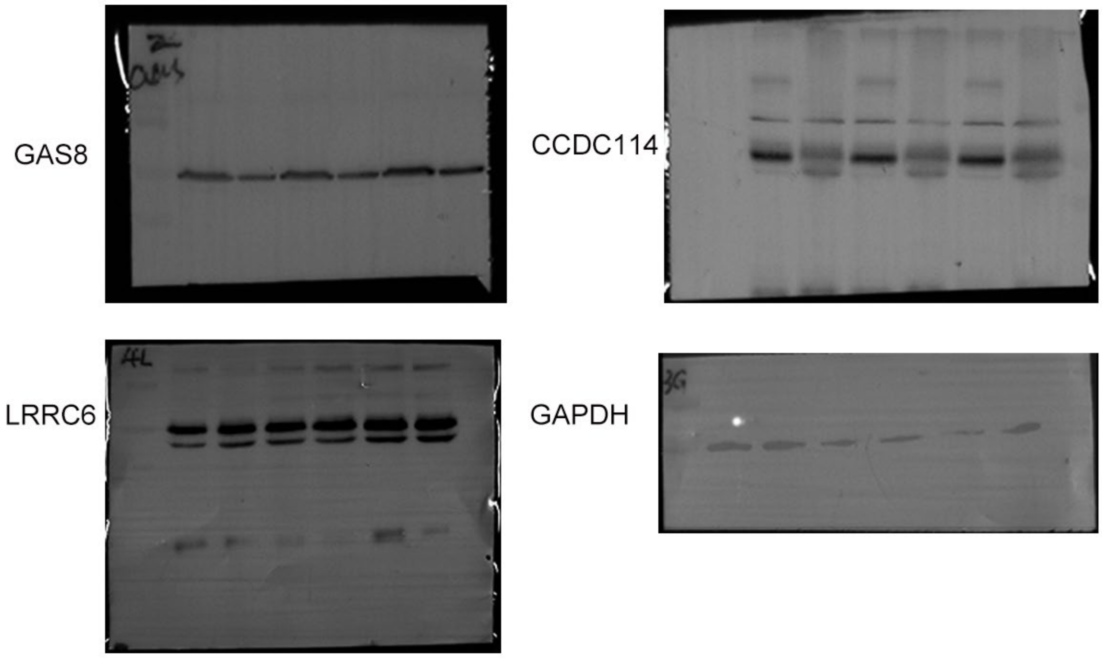

Figure 6D

From left to right, the sample order is Input, igG, and IP-DNAH9

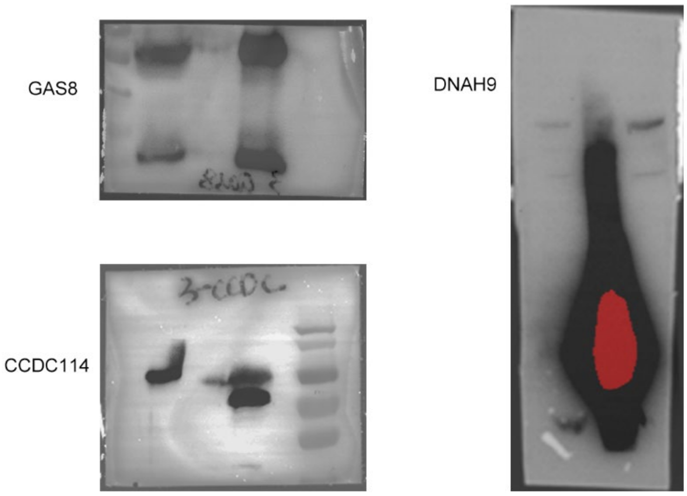

Figure 8E

From left to right, the sample order is WT, *Dnah9* KD, ATP 10μM, ATP 50μM, NADH 10μM, NADH25μM, and NADH 50μM.

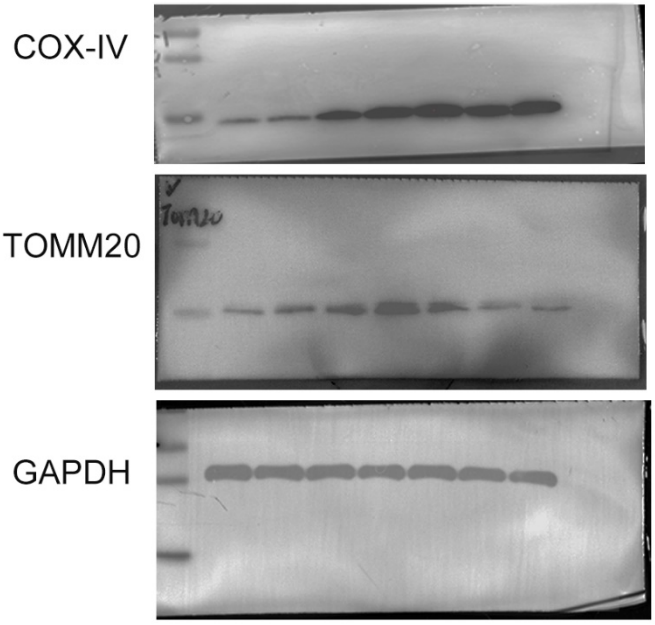

Supplement: Supplementary file 10 — Western blot raw data [file 41419_2022_5010_MOESM10_ESM.pdf]
